# Association between dietary pattern and risk of cardiovascular disease among adults in the Middle East and North Africa region: a systematic review

**DOI:** 10.3402/fnr.v59.27486

**Published:** 2015-06-17

**Authors:** Najlaa Aljefree, Faruk Ahmed

**Affiliations:** 1 Public Health, School of Medicine, Griffith University, Southport, Australia; 2 Menzies Health Institute Queensland, Griffith University, Southport, Australia

**Keywords:** coronary heart disease, stroke, dietary patterns, food items, obesity, diabetes, hypertension, metabolic syndrome, the Middle East, North Africa

## Abstract

**Objective:**

This paper reviews the evidence related to the association of dietary pattern with coronary heart disease (CHD), strokes, and the associated risk factors among adults in the Middle East and North Africa (MENA) region.

**Methods:**

A systematic review of published articles between January 1990 and March 2015 was conducted using Pro-Quest Public Health, MEDLINE, and Google Scholar. The term ‘dietary pattern’ refers to data derived from dietary pattern analyses and individual food component analyses.

**Results:**

The search identified 15 studies. The available data in the MENA region showed that Western dietary pattern has been predominant among adults with fewer adherences to the traditional diet, such as the Mediterranean diet. The Western dietary pattern was found to be associated with an increased risk of dyslipidaemia, diabetes, metabolic syndrome (MetS), body mass index (BMI), and hypertension. The Mediterranean diet, labelled in two studies as ‘the traditional Lebanese diet’, was negatively associated with BMI, waist circumference (WC), and the risk of diabetes, while one study found no association between the Mediterranean diet and MetS. Two randomised controlled trials conducted in Iran demonstrated the effect of the dietary approach to stop hypertension (DASH) in reducing metabolic risk among patients with diabetes and MetS. Likewise, the consumption of dairy products was associated with decreased blood pressure and WC, while the intake of whole grains was associated with reduced WC. In addition, the high consumption of black tea was found to be associated with decreased serum lipids. The intake of fish, vegetable oils, and tea had a protective effect on CHD, whereas the intake of full-fat yoghurt and hydrogenated fats was associated with an increased risk of CHD.

**Conclusion:**

There appears to be a significant association of Western dietary pattern with the increased risk of CHD, strokes, and associated risk factors among adults in the MENA region. Conversely, increased adherence to Mediterranean and/or DASH dietary patterns or their individual food components is associated with a decreased risk of CHD and the associated risk factors. Therefore, increasing awareness of the high burden of CHD and the associated risk factors is crucial, as well as the need for nutrition education programs to improve the knowledge among the MENA population regarding healthy diets and diet-related diseases.

Cardiovascular disease (CVD) is a principal cause of death and disability worldwide; with the number of CVD mortalities increasing globally from 14.4 million in 1990 to 17.5 million in 2005, in particular from coronary heart disease (CHD) and strokes (1). The shift in dietary patterns and the development of nutrition transitions characterised by changes in food supply and intake has been one of the major factors in the high prevalence of CHD and strokes around the world. Numerous factors have contributed to the nutrition transition phenomenon all over the world. Globalisation, a key factor, has had a major effect on changes in lifestyle, food production, modern food processing, and marketing. Furthermore, other factors such as urbanisation, cultural changes, economic development, social improvement, global mass media, and industrialisation have led to predictable shifts in diet and lifestyle (2).

The Middle East and North Africa region (MENA) includes 19 countries. The five countries included from North Africa are Algeria, Egypt, Morocco, Libya, and Tunisia, and the 14 countries included from the Middle East are Bahrain, Iran, Iraq, Israel, Jordan, Kuwait, Lebanon, Oman, Palestine, Qatar, Saudi Arabia, Syria, the United Arab Emirates, and Yemen. The MENA region has almost 355 million people; however, only 8% live in high-income countries (the six Arabic Gulf countries) where the gross domestic product (GDP) per capita is more than US$12,976, while 7% live in low-income countries, such as Yemen, where the GDP per capita is less than US$1,005. The rest of the MENA population (85%) lives in middle-income countries where the GDP per capita is between US$1,006 and US$12,795, such as Iran, Lebanon, Algeria, and Egypt (2, 3).

The MENA region is facing high development and urbanisation, which has resulted in an explosion in the prevalence of CHD and strokes, the key forms of CVD, and the associated risk factors. The mortality rates of ischemic heart disease and cerebrovascular disease (stroke) are estimated to triple between 1990 and 2020 in the majority of MENA countries (4). Several countries in the region have also reported high proportions of CVD mortality. For example, studies from Lebanon and Syria have reported that CVD contributes to 60% and 45% of the total mortality, respectively (5, 6). Furthermore, a high prevalence of CHD has been reported in several countries in the MENA region. For instance, one study from Iran reported 12.7% prevalence of CHD in its study population (7). Similar findings have been reported in studies from Jordan (5.9%), Tunisia (men, 12.5%; women, 20.6%), and Saudi Arabia (5.5%) (8–10).

The MENA region is also witnessing alarming rates of CVD risk factors exceeding those in developed countries. The International Diabetes Foundation (IDF) showed that in 2011 the MENA region had the highest prevalence of diabetes (12.5%) compared to other regions worldwide such as Europe (6%) and Southeast Asia (8.6%) (11). Likewise, the World Health Organization (WHO) data has revealed a significant increase in the prevalence of CVD risk factors within MENA countries, especially obesity which is responsible for almost 30–40% of CVDs (Fig. 1) (12).

**Fig. 1:**
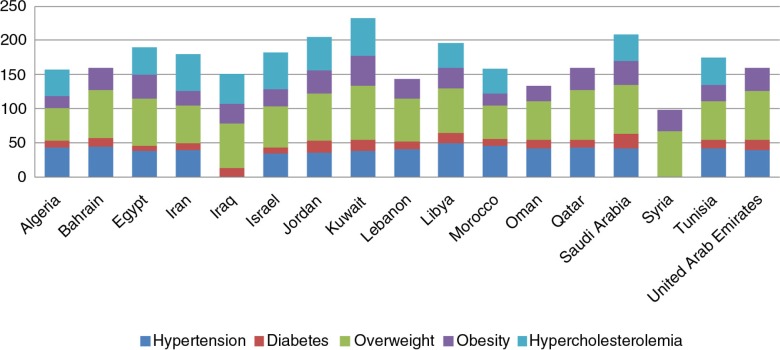
The burden of CHD risk factors (%) in the Middle East and North Africa countries in 2010. Data adopted from World Health Organization (12).

A number of studies have also been published related to the association between diet and chronic diseases in the MENA region. Nevertheless, only one systematic review focusing on nutrition transition and the burden of CVD risk factors in the MENA region has been published (13); however, the association between diet and the presence of CHD and strokes in the region was not examined. Therefore, this paper attempts to fill this gap and review the available literature related to the association of dietary patterns with CHD, strokes, and associated risk factors among adults in MENA countries.

## Methods

### Data sources

Literature searches were conducted using Pro-Quest Public Health, MEDLINE, and Google Scholar to identify both observational studies and randomised controlled trials (RCTs) that were published in English between January 1990 and March 2015. The reference lists of the original articles were also manually searched to identify any further relevant studies. The review articles were also checked. The search terms are shown in Box 1.

Box 1 **Selected search terms**

Dietary patterns

 1. ‘food consumption patterns’ OR ‘dietary patterns’ OR ‘food habits’ OR ‘eating patterns’ OR ‘food items’ OR ‘diet’

The MENA region

 2. ‘The MENA region’ OR ‘the Middle East’ OR ‘North Africa’ OR each country individually

Cardiovascular disease

 3. ‘cardiovascular disease’ OR ‘coronary heart disease’ OR ‘cardiovascular patients’ OR ‘myocardial infarction’ OR ‘coronary artery disease’ OR ‘stroke’ OR ‘cerebrovascular disease’

Associated risk factors

 4. ‘diabetes mellitus’ OR ‘NIDDM’ OR ‘hypertension’ OR ‘high blood pressure’ OR ‘metabolic syndrome’ OR ‘dyslipidaemia’ OR ‘hypercholesterolemia’ OR ‘high cholesterol’ OR ‘overweight’ OR ‘obesity’ OR ‘BMI’

 5. #1 AND #2 AND #3

 6. #1 AND #2 AND #4

### Selection of studies

**Fig. 2:**
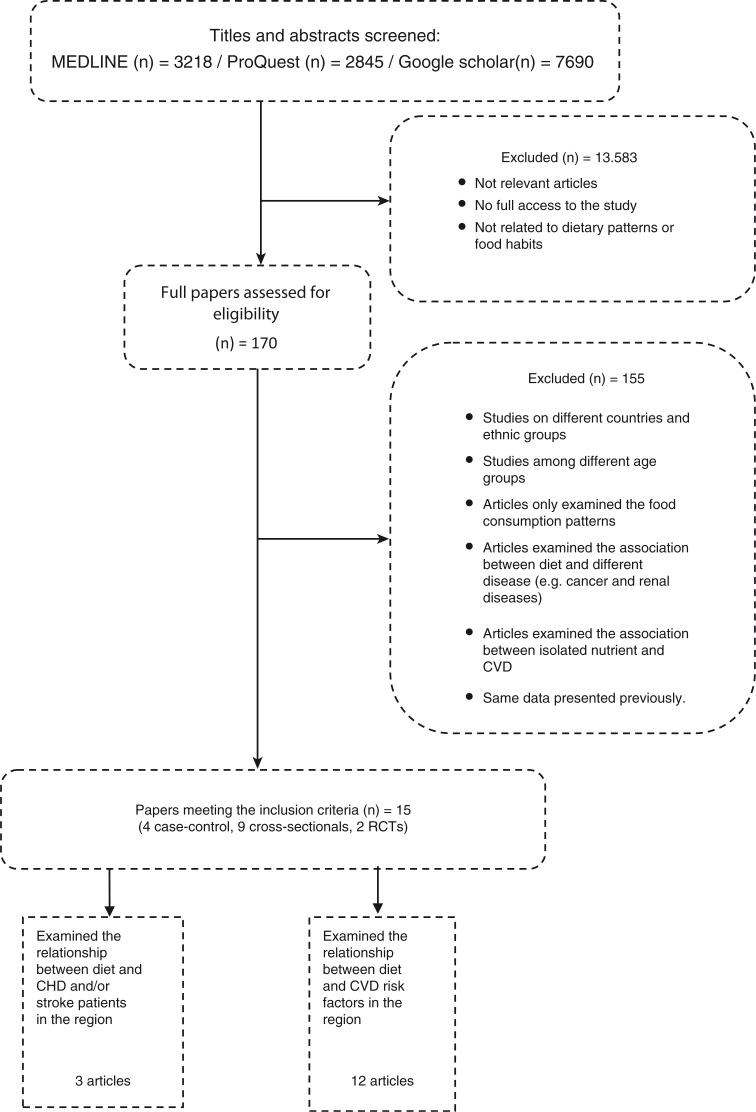
The selection process of the included articles.

The search strategy used certain inclusion criteria that included studies that examined the association of dietary pattern with CHD and/or stroke and/or at least one of the related risk factors: hypertension, diabetes, dyslipidaemia, metabolic syndrome (MetS), overweight, and obesity in the MENA countries. The term ‘dietary pattern’ refers to data derived from either dietary pattern or individual food component analyses. All the included studies were required to only include individuals aged 18 above. All types of populations and socio-demographic backgrounds were included. Furthermore, the review was limited to studies published in English. If the study was carried out among both adults and adolescents or children, only the data on the adult participants were presented. Studies that only reported the prevalence of CHD, strokes, or related risk factors without reporting their association with diet were also excluded. Similarly, this review also excluded studies that only examined the food consumption patterns or food habits without reporting their association with the risk of CHD or strokes or associated risk factors. Studies that only linked isolated nutrients with CHD or strokes or associated risk factors were also excluded. The method to assess which studies were appropriate was based on a hierarchical approach: searching titles, abstracts, and then the full-text papers. Figure 2 shows the selection process.

### Data abstraction and the quality assessment

Data extracted from each study included the following: the country of study, publication year or survey year, study design, the age and gender of the study participants, sample size, a dietary assessment tool, the definition of dietary patterns, diagnosis criteria of CHD, stroke, and related risk factors, the main outcomes and the strengths and limitations of the study. The quality of the included studies was assessed according to the Research Triangle Institute-University of North Carolina, Evidence-based Practice Centre (RTI-UNC EPC) for RCTs, and according to hierarchies of evidence and critical appraisal check list for observational studies (14, 15). The quality assessment of included studies in the systematic review is shown in Tables 1, 2, and 3.

**Table 1:** Quality criteria summary for RCTs studies on the association of dietary patterns with CHD, strokes, and associated risk factors

	Domains									
	
Study	Study question	Study population	Randomisation	Blinding	Interventions	Outcomes	Statistical analysis	Results	Discussion	Funding/support
(24)	Yes	Yes	Yes	Partial	Yes	Yes	Yes	Yes	Yes	Yes
(28)	Yes	Yes	Yes	Partial	Yes	Yes	Yes	Yes	Yes	Yes

Adapted from the Research Triangle Institute–University of North Carolina, Evidence-based Practice Centre (RTI–UNC EPC) for randomized control trials (RCTs) (15).

**Table 2:** Domains and elements for RCTs studies

Domains	Elements
Study question	• **Clearly focused and appropriate question**
Study population	• **Description of study population** • **Specific inclusion and exclusion criteria** • Sample size justification
Randomisation	• Adequate approach to sequence generation • **Adequate concealment method used** • Similarity of groups at baseline
Blinding	• **Double-blinding (e.g. of investigators, caregivers, subjects, assessors, and other key personnel as appropriate) to treatment allocation**
Interventions	• **Intervention(s) clearly detailed for all study groups (e.g. dose, route, timing for drugs, and details sufficient for assessment and reproducibility for other types of interventions)** • Compliance with intervention • Equal treatment of groups except for intervention
Outcomes	• **Primary and secondary outcome measures specified** • Assessment method standard, valid, and reliable
Statistical analysis	• **Appropriate analytic techniques that address study withdrawals, loss to follow-up, missing data, and intention to treat** • Power calculation • Assessment of confounding • Assessment of heterogeneity, if applicable
Results	• **Measure of effect for outcomes and appropriate measure of precision** • Proportion of eligible subjects recruited into study and followed up at each assessment
Discussion	• **Conclusions supported by results with possible biases and limitations taken into consideration**
Funding or sponsorship	• *Type and source of support for study*

Elements appearing in italics are those with an empirical basis. Elements appearing in bold are those considered essential to give a system a Yes rating for the domain. Adapted from the Research Triangle Institute–University of North Carolina, Evidence-based Practice Centre (RTI–UNC EPC) for randomized control trials (RCTs) (15).

**Table 3:** Quality criteria summary for the observational studies on the association of dietary patterns with CHD, strokes, and associated risk factors in the MENA region

Study	Explicit aims	Sample size justification or adequate	Sample representative of population	Inclusion and exclusion criteria stated	Reliability and validity of measures justified	Response rate and drop out specified	Data adequately described	Statistical significance assessed	Discussion of generalisability
(17)	Yes	Yes	No	Yes	Yes	Yes	Yes	Yes	No
(16)	Yes	No	No	In part	No	Yes	Yes	Yes	No
(26)	Yes	Yes	No	Yes	Yes	No	Yes	Yes	No
(22)	Yes	Yes	No	Yes	Yes	No	Yes	Yes	Yes
(30)	Yes	Yes	No	Yes	Yes	Yes	Yes	Yes	Yes
(29)	Yes	Yes	Yes	No	Yes	No	Yes	Yes	No
(31)	Yes	Yes	No	Yes	No	No	Yes	Yes	No
(23)	Yes	Yes	No	Yes	No	Yes	Yes	Yes	In part
(27)	Yes	Yes	No	Yes	Yes	No	Yes	Yes	No
(25)	Yes	Yes	No	No	Yes	Yes	Yes	Yes	No
(18)	Yes	Yes	No	Yes	Yes	No	Yes	Yes	No
(19)	Yes	Yes	No	Yes	Yes	Yes	Yes	Yes	No
(20)	Yes	Yes	No	Yes	Yes	Yes	Yes	Yes	No

NA: Not applicable. Adapted from the hierarchies of evidence and critical appraisal check list (14).

### Data synthesis

The analysis of included studies involved a narrative synthesis to examine the objective of this review due to the non-homogenous nature of included studies. The synthesis started with an initial summary of the main characteristics and outcomes of included studies through organised tables and assessed the strength and limitations of studies. It also included a clear description of the papers in the review and a summary of the main results, and considered the association between individual studies and between the findings of diverse studies.

## Results

The literature search identified 15 studies that met the inclusion criteria and were all from the Middle East: six papers were published in the 2000s and nine papers in the last 4 years. Of the included studies, three studies evaluated the dietary patterns and/or specific food items among CHD and stroke patients, and 12 studies examined the association of dietary patterns and/or specific food items with CVD risk factors in the MENA region. Of the studies that reported the association between CVD risk factors and dietary patterns, three investigated diabetes, five focused on MetS, one examined overweight and obesity, one addressed blood lipids and three investigated multiple risk factors.

Out of the 15 included articles, 10 were located in Iran, 3 in Lebanon, and 2 in Saudi Arabia. There was a lack of data from the majority of the MENA. Regarding the study designs, four were case–control, nine were cross-sectional, and two were RCTs. Furthermore, the main populations included in this review were university students (one study), patients (six studies), and the general population (eight studies). The sample sizes in the included studies ranged from 31 to 1,764 subjects and the response rates ranged from 24.3% (16) to 95.9% (17). Details of the research methods and key findings of the included articles are summarised in Tables 4 and 5.

**Table 4:** Summary of characteristics and main findings from the included studies examined the dietary patterns among CHD and stroke patients in the MENA countries

Reference, country and survey year	Sample size and gender proportion	Age groups	Study design and sampling methods	Factors studied	Diagnostic criteria	Dietary assessment methods	Main findings	Strength and limitations of included studies
(20)/Iran/2008	Stroke patients: 195 M: 60% and F: 40% Control group: 195 M: 49% and F: 51%	Stroke patients (66.9 years) Control subjects (60.8 years)	Case–control study/convenience non-random sample selection for cases and control subjects	The association between the intake of SSBs and risk of stroke	Ischaemic stroke defined as an episode of focal neurologic deficit with acute onset due to a vascular cause and lasting more than 24 h	A validated semi-quantitative FFQ with 168 food items	• There were no statistical differences between stroke patients and control group in the mean consumption of sugar-sweetened beverages (48.2 vs. 47.2 g/day, *p*=0.90) even after the adjustment of confounding factors.	• Noted limitations were the failure to match stroke patients and the control group, as the latter group was older. Further, because of the inability of stroke patients to remember their food intake, the family members of the patients completed the FFQ. • However, it was the first study among stroke patients in the Middle East and had a high response rate (93%)
(19)/Iran/2008				The association between potato consumption and risk of stroke			• The mean consumption of whole-fat dairy, pulses, potato and fruits was significantly higher among stroke patients in comparison to the control group, (132.2 vs. 73.6 g/day, *p*<0.001), (34.6 vs. 25 g/day, *p*<0.001), (31.1 vs. 23.4 g/day, *p*<0.05), and (358.6 vs. 280.5 g/day, *p*<0.05). • The mean consumption of low-fat dairy and vegetable oils was significantly lower among stroke patients than the control group, (270.3 vs. 339.9 g/day, *p*<0.05) and (10.4 vs. 19.2 g/day, *p*<0.001) respectively. • A significant positive association between the consumption of potatoes and the risk of strokes has been proved after an adjustment for confounding factors (OR=1.9, 95% CI: 1.0–3.6)	
(18)/Iran/2004–2006	CHD patients: 108 M: 53% and F: 47%	CHD patients (51.5 years)	Case–control study/random selection of CHD and control subjects	The comparison in dietary pattern between CHD	CHD patients (more than 70% stenosis in each of the main coronary vessels or subjects	A semi-quantified validated FFQ with 41 food items	• The consumption of hydrogenated fats and whole-fat yoghurt was significantly associated with the increased risk of CHD (OR=2.12, 95% CI: 1.23–3.64) and (OR=2.35, 95% CI: 1.32–4.18) respectively.	• The limitation was the risk of bias in selecting the control subjects.
	Control: 108 M: 52% and F: 48%	Control (50.8 years)	from the catheterisation wards from two hospitals	patients and control group	with myocardial infarction). Control group (subjects with atypical chest pain and had normal angiography)		• The consumption of fish, vegetable oils and black tea on the daily basis was significantly associated with a decreased risk of CHD (OR=0.55, 95% CI: 0.31–0.91), (OR=0.23, 95% CI: 0.13–0.42), and (OR=0.3, 95% CI: 0.15–0.65) respectively.	
							• There were no significant associations between the risk of CHD and the consumption of red meat, chicken and eggs on the weekly basis as well as the consumption of fruits and vegetables on the daily basis.	

M: male; F: females; CHD: coronary heart disease; FFQ: food frequency questionnaire; g: gram; NR: not reported.

**Table 5:** Summary of characteristics and main findings from the included studies examined the association between diet and CVD risk factors in the MENA countries

Reference, country and survey year	Sample size and gender proportion	Age groups	Study design, sampling methods and response rate (%)	Factors studied	Diagnostic criteria	Dietary assessment methods	Main findings	Strength and limitations of included studies
(23)/Lebanon/2009–2010	T2D patients: 58 Control: 116 M: 60.3% and F: 39.7% in both groups	T2D (56.5 years) Control (55.9 years)	Case–control study/convenience non-random sample selection for cases and random selection for control/89% for cases and 82% for control	The association between dietary patterns and the odds of T2D among newly diagnosed patients	NR	Semi-quantitative FFQ with 97 food items	• 4 main dietary patterns identified using factor analysis: Refined grains and desserts (rich in pastas, pizza, white bread, and desserts), Traditional Lebanese (rich in olives oil, fruits and vegetables, whole wheat bread, and traditional dishes), fast food (rich in French fries, fast-food sandwiches, mixed nuts, and whole fat diary), and Meat and alcohol pattern (rich in eggs, alcohol, read meats, and sweetened beverages). • The refined grains and desserts pattern and the fast-food pattern were associated with the increase risk of type 2 diabetes (OR=3.85, 95% CI: 1.13–11.23) and (OR=2.80, 95% CI: 1.41–5.59) respectively after adjustment of confounding factors • The traditional Lebanese dietary pattern was associated with decrease risk of type 2 diabetes (OR=0.46, 95% CI: 0.22–0.97). • The refined grains and desserts, the fast food, and the meat and alcohol patterns were positively associated with BMI and WC • The refined grains and desserts pattern had the highest variance (13.68%) followed by the traditional Lebanese (10.8%) and fast food (9.13%).	• The FFQ used in this survey was not validated; however it was completed by a qualified dietician and not self-reported. • The risk of recall bias and the possibility of changing the food intake because of medical advice and relatively small sample size were the limitations
(24)/Iran/2009	31 T2D patients M: 13 and F: 18	NR	Randomised crossover design/random selection	The effect of DASH diet on the metabolic risk in T2D patients	T2D defined as FPG ≥126 mg/dl or on medication	The 3-day food diaries	• After 8 weeks period, DASH diet was significantly associated with the reduction in weight and WC. • the mean changes for fasting blood glucose, LDL cholesterol, TC, systolic blood pressure (SBP) and diastolic blood pressure (DBP) were reduced after DASH eating pattern (−29.4 mg/dl), (−17.2 mg/dl), (−22.1 mg/dl), (−13.6 mmHg), and (−9.5 mmHg) respectively. • The mean change for HDL cholesterol was increased (4.3 mg/dl) after DAH diet.	• major strength is the use of RCT design and thus provided strongest evidence
(22)/Iran/2003–2008	425 IGT subjects M and F: NR	35–55 years	Cross-sectional study/convenience sampling	The association between dietary patterns and MetS among subjects with IGT	MetS defined according to ATP III criteria	FFQ with 39 food items	• 5 dietary patterns were identified: Western pattern, prudent pattern, vegetarian pattern, high-fat dairy pattern, and chicken and plant pattern. • After adjustment for the confounding variables, the Western pattern (rich in sugar, butter, soda, sweets, eggs, hydrogenated fat, and mayonnaise) was significantly associated with increased levels of triacylglycerol and blood pressure (OR=1.76, 95% CI: 1.01–3.07) and (O=2.62, 95% CI: 1.32–5.23) respectively. • The vegetarian pattern (rich in green leafy vegetables, fruits, potatoes and legumes) was associated with increased plasma glucose levels (OR=2.26, 95% CI: 1.25–4.06).	• A noted limitation was the use of a short FFQ with 39 food items • The FFQ did not separate the questions regarding refined from whole grain; thus the dietary analysis did not separate the consumption of refined and whole grain.
(27)/Iran	827 M: 357 and F: 470	18–74 years	Cross-sectional study/multistage cluster random sampling	The association between dairy consumption and MetS	MetS defined according to ATP III criteria	Semi quantitative FFQ with 168 food items	• Subjects in quartile 4 (highest) of the dairy intake (milk, yoghurts, and cheese) had significantly lower mean WC, SBP and DBP than subjects in quartiles 1 (lowest), (76 vs. 81 cm), (112 vs. 128 mmHg), and (83 vs. 89 mmHg) respectively. • After adjustment for the confounding variables, subjects in the highest quartile of dairy intake had lower odds of getting MetS (OR by quartile: 1, 0.83, 0.74, 0.69, *P*<0.02).	• The survey used a population-based sample; however, it was representative of a large city (Tehran) in Iran but not the entire population in Iran.
(17)/Iran	984 F: 100%	30–50 years	Cross-sectional study/systematic random sampling/(95.9%)	The association between diet (components of various food groups) and MetS among middle aged women	MetS defined according to ATP III criteria	Validated FFQ	• By comparing females with MetS (*n*=284) with females without MetS (*n*=632), females with MetS showed a significantly lower consumption of meat, fruits, vegetables, cereals, dairy products, oil and butter and a significantly higher consumption of bread and grains than females without MetS. • Carbohydrates rather than fats contributed a lot more to the total energy intake of females with MetS compared to females without MetS. • 4 food components identified: 1 a healthy food pattern, 2 a high glycaemic index and high-fat pattern, 3 a pattern include the intake of pasta, 4 the dairy products and eggs, 5 the pattern include the intake of sweets. • Food component 1 and 4 were associated with the reduce risk of MetS, while food component 3 was positively associated with increased blood lipids.	• The main strength of the survey was the systematic random sampling used to select the study subjects; however, they were only derived from an urban population
(16)/Lebanon/2008–2009	323 M: 160 F:163	≥18 years	Cross-sectional study/multistage random sampling/(24.3%)	The association between various dietary patterns and risk of MetS	MetS defined according to the International Diabetes Federation	FFQ with 61 food items	• 3 dietary patterns identified using factor analysis: fast-food and desserts pattern, traditional Lebanese pattern, and high-protein pattern. • The variance of the fast-food and desserts pattern was the highest compared to the traditional and high-protein patterns (13.11, 9.71, and 7.8% respectively). • The fast-food and desserts pattern (rich in fast-food sandwiches, pizzas, desserts, soft drinks) was associated with an increased risk of MetS (OR=3.13, 95% CI: 1.36–7.22) and hyperglycaemia (OR=3.81, 95%CI: 1.59–9.14). • The high-protein pattern (rich in chicken, fish, low-fat dairy products, meats) was associated with an increased risk of high blood pressure (OR=2.98, 95% CI: 1.26–7.02). • There was no association between the traditional Lebanese pattern (rich in olives, fruits, legumes, vegetable oil, grains) and the risk of MetS.	• The main limitation was the low response rate (24.3%). • The FFQ used in the study was not validated; however, the data was collected by a qualified dietician and no self-reported
(26)/Iran	827 M: 357 and F: 470	18–74 Years	Cross-sectional study/multistage cluster random sampling	The association between whole-grains intake and HW in adults	The WC cut-offs values used were 80 cm for males and 79 cm for females. For the serum triacylglycerol concentrations, the triacylglycerol ≥150 mg/dl used as the cut-off based on the NCEP ATP III recommendations.	Semi quantitative FFQ with 168 food items	• Subjects in the highest quartile of the consumption of whole-grains (including all dark breads such as Iranian bread Tafton, barbari and sangak breads as well as barley bread, popcorn and Iranian whole grain cornflakes) had significantly lower rates of HW (29%) than subjects in the lowest quartile (44%). • Subjects in the highest quartile of the consumption of refined grains (biscuits, white breads, French bread, noodles, pasta and rice) had significantly higher rates of HW (45%) than subjects in the lowest quartile (27%). • After adjustment for the confounding variables, a significant negative association between whole-grains intake and WC was found, while the association between refined-grains and WC was positive but not significant. • Participants who consumed large amounts of whole-grain products were also consumed large amounts of fruits, vegetables and fibre, and less consumed meat and cholesterol.	• A noted limitation was the accurate separation between whole-grains and refined grains was difficult because of the fixed food categories in the FFQ used
(28)/Iran	116 MetS patients M: 34 and F: 82	41.2 years	Randomised controlled trial	The effect of DASH diet on patients with MetS	MetS defined according to the International Diabetes Federation	The 3-day food diaries	• After a 6 month intervention, the DASH diet had more positive effects on the reduction of the metabolic risks in MetS patients compared to the weight-reducing diet. • The mean change in WC (M: 98, F: 90; M: 100, F: 91 cm), weight (M: 71, F: 57; M: 73, F: 58 kg), triglycerides (M: 185, F: 217; M: 187, F: 216 mg/dl), SBP (M: 133, F: 132; M: 136, F: 141 mmHg), and DBP (M: 81, F: 78; M: 87, F: 83 mmHg) was significantly lower among male and female subjects on the DASH diet than those on the weight-reducing diet respectively.	• Strength of evidence is strong as the study used RCT design.
(25)/Lebanon/2005	787 M: 52%, F: 48%/R 100%	40–60 years	Cross-sectional study/random selection/(85%)	The relation between Mediterranean diet and obesity among rural population	Overweight and obesity defined according to the WHO criteria	Non-quantitative FFQ and a 24-h recall	• A low adherence to Mediterranean diet was found among the study population as it partly matched the traditional Mediterranean diet. • The Mediterranean diet score (MDS) was inversely associated with WC but not BMI in both genders. • The compound Mediterranean score for both positive factors of the Mediterranean pattern (fruits, vegetables, legumes, olive oil and whole cereals) as well as negative factors adopted by the study subjects (pastries, liquid sweets and refined cereals) was negatively associated with WC and BMI in both genders.	• Noted limitations were the limited sample size and use of a qualitative FFQ, however, it was combined with 24-h recall
(29)/Saudi Arabia/1993–1998	1,764 females	30–70 years	Cross-sectional study/multistage stratified cluster sampling	The association between black tea consumption and serum lipids and lipoproteins	NR	Structured questionnaire	• The daily consumption of black tea was reported among 87.2% of the study subjects. • Saudi females who daily consumed more than six cups of tea (>480 ml) were significantly more likely than those who did not, to have lower rates of dyslipidaemia including, high (TC) (OR=0.63, 95% CI: 0.41–0.97), high triglycerides (OR=0.56, 95% CI: 0.35–0.86), high (LDL) (OR=0.70, 95% CI: 0.45–1.07), and high (VLDL) (OR=0.61, 95% CI: 0.39–0.93). • There was no association between black tea consumption and (HDL).	• Misclassification of the intake of beverage might be a limitation. • The large representative sample from the National survey was a strength of the study
(31)/Saudi Arabia/2008–2009	312 students M:132, F:180	21.1 years	Cross-sectional study/random selection/	The association between overweight and obesity and HTN and dietary habits	BMI defined according to the National Institute of Health. HTN defined according to the Fourth Report on the Diagnosis, Evaluation, and Treatment of High Blood Pressure in Children and Adolescents.	Self-reported questionnaire (11 items)	• The proportion of total energy from carbohydrates and fats was very high especially from fats (38% vs. 39%) and (46.1% vs. 46.8%) in both males and females. • The proportion of total energy from protein was (17% vs. 15%) in males and females. • A high intake of mono-unsaturated fats and saturated fats was reported as 21 and 14% in both genders, whereas the mean intake of fibre was low in both genders. • Some unhealthy food habits were reported including a high consumption of snacks (daily) in almost 42.5%, a low consumption of vegetables (1 to 2 times weekly) in 30%, a high consumption of fatty foods (3–4 times weekly) in 36% of the females and 44% of the males, a high consumption of salty foods (daily) in 36% of the females and 43% of the males and a high consumption of sugar (daily) in 41% of the females and 38% of the males. • A significant association between the high intakes of energy derived from fatty foods and BMI and hypertension in both genders. Similarly, a significant association was found between the high consumption of salty foods and hypertension. • A negative association was found between the consumption of vegetables, grains and beans and BMI and hypertension in both genders.	• The study sample was derived from one university in Riyadh city, which makes it hard to generalise the results to wider population in Saudi Arabia.
(30)/Iran	486 F: 100%	40–60 years	Cross-sectional study/multistage cluster random sampling/(89%)	The association between dietary patterns and CVD risk factors	Dyslipidaemia was defined based on the third report of the National Cholesterol Education Program Expert Panel. HTN: SBP ≥ 140 mm Hg or DBP ≥ 90. T2D: fasting blood glucose ≥6.93 mmol/l	Validated semi-quantitative FFQ with 168 food items	• The study identified three main dietary patterns using principle component analysis: healthy pattern (rich in fruits, vegetables, legumes, fruit juices, poultry, whole grains), Western pattern (rich in red meat, high-fat dairy products, refined grains, hydrogenated fats, sweets, soft drinks, eggs, pizza), and Iranian pattern (rich in potato, tea, refined grains, whole grains, legumes, hydrogenated fats). • After controlling for confounders, the healthy dietary pattern was significantly associated with decreased levels of dyslipidaemia (OR=0.36, 95% CI: 0.19–0.53), HTN (OR=0.33, 95% CI: 0.17–0.60), and at least 2 risk factors (OR=0.39, 95% CI: 0.20–0.77).	• The main limitation of this survey was that it was conducted among only females and the sample derived from only Tehran city so it is difficult to generalise the results across all Iranian females.
							• The Western pattern was significantly associated with increased dyslipidaemia (OR=2.59, 95% CI: 1.41–4.76), HTN (OR=2.61, 95% CI: 1.27–5.19), and at least 2 risk factors (OR=2.65, 95% CI: 1.20–5.64). • The Iranian pattern was only significantly associated with increased dyslipidaemia (OR=1.73, 95% CI: 1.02–2.99). • There was no association between the three dietary patterns and T2D.	

M: male; F: females; R: rural; CVD: cardiovascular disease; CHD: coronary heart disease; T2D: type 2 diabetes; BMI: body mass index; WC: waist circumference; FPG: fasting plasma glucose; LDL: low-density lipoprotein; HDL: high-density lipoprotein; VLDL: very low-density lipoprotein; TC: total cholesterol; HC: hypercholesterolemia; SBP: systolic blood pressure; DBP: diastolic blood pressure; Mets: metabolic syndrome; IGT: impaired glucose tolerance; HW: hypertriglyceridemia waist; HTN: hypertension; FFQ: food frequency questionnaire; g: gram; cm: centimetre; kg: kilogram; WHO: World Health Organization; DASH: dietary approaches to stop hypertension; NR: not reported.

### Association of dietary patterns with CHD and strokes in the MENA region

There were only three case–control studies that have examined the dietary intake among CHD (18) and stroke patients in the MENA region (19, 20), all of which were carried out in Iran. A summary of these studies is shown in Table 4. The studies examined the food consumption patterns among CHD and stroke patients and compared them with matched controls to determine whether the intake of various food items was associated with the risk of CHD and strokes. In CHD patients, the daily intake of fish, vegetable oils, and tea had a protective effect on CHD, whereas the daily intake of full-fat yoghurt and hydrogenated fats was positively associated with an increased risk of CHD (18). In stroke patients, the consumption of potatoes was associated with an increased risk of stroke (19), while no association was found between the intake of sugar-sweetened beverages and the risk of stroke (20). In addition, when compared to the control group, stroke patients had a higher consumption of high-fat dairy, pulses, and fruits and a lower consumption of low-fat dairy and non-hydrogenated vegetable oils (19).

### Association between dietary patterns and CVD risk factors in the MENA region

In order to clarify the association between diverse dietary patterns and the risk of CHD and strokes, it is crucial to examine the effect of the dietary intake on various risk factors such as blood glucose levels, blood lipids level, blood pressure, and body weight (21). Twelve studies have examined the relationship of dietary patterns and/or individual food items with CVD risk factors among adults in the MENA countries. The details of the methodologies and key findings of the included studies are summarised in Table 5.

#### Type 2 diabetes

A cross-sectional survey among Iranian adults with Impaired Glucose Tolerance (IGT) found that a Western dietary pattern (rich in sugar, butter, soda, sweets, eggs, hydrogenated fat, and mayonnaise) was significantly associated with increased levels of triacylglycerol (OR=1.76, 95% CI: 1.01–3.07) and blood pressure (OR=2.62, 95% CI: 1.32–5.23), while a vegetarian dietary pattern (rich in green leafy vegetables, fruits, potatoes, and legumes) was associated with increased plasma glucose levels (OR=2.26, 95% CI: 1.25–4.06) after adjustment for the confounding variables (22).

Another case–control study conducted among type 2 diabetes patients in Lebanon using factor analysis identified four main dietary patterns: refined grains and desserts (rich in pastas, pizza, white bread, and desserts), a traditional Lebanese diet (rich in olives oil, fruits and vegetables, whole wheat bread, and traditional dishes), fast food (rich in French fries, fast-food sandwiches, mixed nuts, and whole fat diary), and meat and alcohol pattern (rich in eggs, alcohol, read meats, and sweetened beverages) (23). This study showed, after adjustment for confounding factors, that the refined grains and dessert dietary pattern (OR=3.85, 95% CI: 1.13–11.23) and fast-food dietary pattern were associated with an increased risk of type 2 diabetes (OR=2.80, 95% CI: 1.41–5.59), whereas, the traditional Lebanese dietary pattern was associated with a decreased risk of type 2 diabetes (OR=0.46, 95% CI: 0.22–0.97) (23). Similarly, a randomised crossover clinical trial conducted among patients with type 2 diabetes in Iran also reported the beneficial effects of the DASH diet on several cardio metabolic risks (24). The study demonstrated that when compared to the control group, there were significant reductions in fasting blood glucose (FBG), low-density lipoproteins (LDL) cholesterol, total cholesterol (TC), and blood pressure levels among the participants who followed the DASH diet for 8 weeks, while there was a significant increase in high-density lipoproteins (HDL) cholesterol level (24).

#### Obesity

A number of studies have supported the fact that the traditional Mediterranean diet is associated with a reduction in overweight and obesity. For example, a cross-sectional survey in Lebanon showed that the Mediterranean diet (whole cereals, legumes, olive oil, fruit, fish, and vegetables) was negatively associated with a high waist circumference (WC) and body mass index (BMI) in both genders (25). Likewise, according to a study conducted by Naja et al. (23), the traditional Lebanese dietary pattern (rich in olives oil, fruits and vegetables, whole wheat bread) was also negatively associated with elevated BMI and WC among type 2 diabetes patients. The same study also reported that the refined grains and desserts dietary pattern, the fast-food dietary pattern, and the meat and alcohol dietary pattern were positively associated with high BMI and WC among type 2 diabetes patients (23).

#### Metabolic syndrome

A study analysing data from a nation-wide survey in Lebanon showed that the fast food and desserts dietary pattern (rich in fast-food sandwiches, pizzas, desserts, soft drinks) was associated with an increased risk of MetS (OR=3.13, 95% CI: 1.36–7.22) and hyperglycaemia (OR=3.81, 95% CI: 1.59–9.14), and the high-protein dietary pattern (rich in chicken, fish, low-fat dairy products, meats) was associated with an increased risk of high blood pressure (OR=2.98, 95% CI: 1.26–7.02) (16). There was no association between the traditional Lebanese dietary pattern (rich in olives, fruits, legumes, vegetable oil, grains), which is similar to traditional local Mediterranean food, and the risk of MetS (16). Another study from Iran reported that a dietary pattern rich in cereals, fruits and vegetables, legumes, fish, eggs, and dairy products was associated with a reduced risk of MetS, while a dietary pattern rich in pasta was positively associated with increased blood lipids (17).

A study conducted among Iranian adults showed that the intake of whole grains was associated with reduced hypertriglyceridemia waist (HW) (serum triacylglycerol concentration and WC) and WC (26). Moreover, another survey in Iran, which examined the relationship between MetS and the intake of dairy products, indicated that the consumption of dairy products was associated with decreased blood pressure and WC (27). Similarly, an RCT conducted in Iran demonstrated a beneficial effect of the DASH diet in reducing the metabolic risk among patients with MetS (28). For example, when compared with the control group, significant reductions in body weight, triglycerides, blood pressure, and FBG levels were observed in both males and females in the DASH diet group (28).

#### Abnormal blood lipids

One study in Saudi Arabia showed that subjects who consumed more than six cups of black tea per day (>480 ml) were significantly less likely to have dyslipidaemia including, high TC (OR=0.63, 95% CI: 0.41–0.97), high triglycerides (OR=0.56, 95% CI: 0.35–0.86), and high very low-density lipoproteins (VLDL) (OR=0.61, 95% CI: 0.39–0.93) (29). However, this study did not find any association between black tea consumption and high LDL and, HDL (29).

#### Multiple CVD risk factors

In Iran, a study found that a healthy dietary pattern (rich in fruits, vegetables, legumes, fruit juices, poultry, whole grains) was significantly associated with a reduced risk of dyslipidaemia (OR=0.36, 95% CI: 0.19–0.53), hypertension (OR=0.33, 95% CI: 0.17–0.60) (30). Conversely, the Western dietary pattern (rich in red meat, high-fat dairy products, refined grains, hydrogenated fats, sweets, soft drinks, eggs, pizza) was significantly associated with an increased risk of dyslipidaemia (OR=2.59, 95% CI: 1.41–4.76), hypertension (OR=2.61, 95% CI: 1.27–5.19) (30). The Iranian dietary pattern (rich in potato, tea, refined grains, whole grains, legumes, hydrogenated fats) was only significantly associated with an increased risk of dyslipidaemia (OR=1.73, 95% CI: 1.02–2.99) (30). In Saudi Arabia, a food consumption survey showed a significant association between the high intake of energy derived from fatty foods and high BMI and hypertension levels in both genders (31). Similarly, a significant association was found between the high consumption of salty foods and hypertension, while a negative association was found between the consumption of vegetables, grains and beans and BMI and hypertension in both genders (31).

## Discussion

This review has revealed that specific dietary patterns and/or the individual food components of a diet are associated with CHD, strokes, and associated risk factors among adults in MENA countries. When looking at the individual food components of a diet, studies have shown that the intake of fish, vegetable oils and black tea had a protective effect on CHD, while the intake of full-fat yoghurt and hydrogenated fats were positively associated with the risk of CHD (18–20). These findings were similar to those reported among the US population (32, 33), except for the effect of black tea on CHD (34). In addition, this review found a significant positive association between potato consumption and the risk of strokes (19), and this has been explained by the high glycaemic index and the high amount of carbohydrates which make potato a type of food that increases the risk of strokes. Similar results have been reported in Australia leading to conclusions that foods with the high amount of glycaemic index, such as potatoes, may increase the risk of stroke death (35).

The MENA region has witnessed vast changes in dietary patterns resulting from the marked shifts in socio-economic status and demographics, as well as rapid urbanisation and modernisation during the last few decades. This review also identified that the Western dietary pattern rich in sweets, fatty foods, meat, whole dairy products, fast food, salty nuts, and canned foods has become predominant among the majority of the MENA population (16, 22, 23, 30). These results are consistent with available food consumption surveys among adults in the MENA region, such as in Lebanon, Egypt and Iran, which have indicated that eating patterns are mainly characterised by a high intake of sugar, meat, soft drinks and refined grain, as well as low consumption of fish, fruits, vegetables, legumes and whole grains (36–38). Further, using a factor analysis of dietary intake data from a national survey in Iran, it has also been reported that the majority of the study subjects were consuming the Western dietary pattern (high in sweets, fast foods, salty nuts, canned foods) (39). These findings were also consistent with the FAO food balance sheets data, which indicated that the availability of sugar and sweeteners (kg/person/year) has gradually increased gradually in the MENA region during the last four decades especially in oil-rich countries such as Saudi Arabia and Algeria (40).

The findings of this review also indicate that adopting the Western dietary pattern is significantly associated with almost all CVD risk factors, including an increased risk of obesity, blood lipids, hypertension, diabetes and MetS in MENA populations (16, 22, 23, 30). Similar results have been reported among US and Mexican populations (41, 42). On the other hand, the Mediterranean diet, also known as the healthy diet, is the traditional diet in North African countries and three Middle Eastern countries, Syria, Israel and Lebanon, and it has been inversely associated with obesity, diabetes, and MetS among the same populations (16, 23, 25). Therefore, there is increasing evidence illustrating the health benefits of the Mediterranean diet in the reduction of morbidity and mortality from CVD (43, 44). The Mediterranean dietary pattern mainly consists of a high consumption of fruits, vegetables, wholegrain cereals, legumes, nuts, olive oil, fish and seafood. It is also characterised by a moderate consumption of dairy products, eggs, poultry and wine (45). These individual food elements offer many benefits for cardiovascular health and prevention of CVD, as they are good sources of monounsaturated fatty acids, antioxidants, magnesium, fibre, and polyphenols (45).

### Limitation of the review

The major limitation in this review was the lack of studies from several countries in the MENA region that have examined eating patterns among CHD and stroke patients; only three case–control studies were identified and all of them were from Iran. In addition, the majority of the included studies in the review utilised cross-sectional design, and thus failed to assess causal relationships. Further, most of the studies reported dietary patterns using food frequency questionnaires which might have increased the risk of recall bias and overestimated the consumption of healthy foods such as fruits and vegetables. Also, some of the studies that reported (16, 23) significant associations between diet and CVD had a very wide confidence intervene for odd ratios (ORs) which may indicate that the results of these studies are not reliable either due to small sample sizes or the sampling procedures are not representative.

## Conclusion

This review demonstrates that Western dietary patterns, which are mainly characterised by high consumption of sugar, fatty foods, meat, and refined grains, and low consumption of fruits, vegetables, fibre, and whole grains, are very common in the MENA region and adherence to this Western diet is linked with an increased risk of CHD, strokes, and associated risk factors among adult populations of the MENA countries. On the contrary, adherence to the Mediterranean diet or DASH diet and/or individual food components of these diets appears to be associated with a decreased risk of CHD and associated factors. Therefore, increasing awareness of the high prevalence of CVD and associated risk factors among the public is crucial. In addition, there is an urgent need for nutrition education programs among all segments of the MENA population to increase awareness regarding healthy diets and diet-related chronic diseases. This should be combined with encouragement of healthy lifestyle patterns, including increasing physical activity and a reduction in smoking, to enhance the prevention of heart disease and associated risk factors. Furthermore, there is a crucial need for further intervention studies focusing on a range of diverse cohorts in relation to CHD and strokes, in order to help in providing nutritional recommendations to control CVD among the population of the MENA region.
